# Relationship between Plasma Lipid Profile and Cognitive Status in Early Alzheimer Disease

**DOI:** 10.3390/ijms25105317

**Published:** 2024-05-13

**Authors:** Carmen Peña-Bautista, Lourdes Álvarez-Sánchez, Gemma García-Lluch, Luis Raga, Paola Quevedo, Mar Peretó, Angel Balaguer, Miguel Baquero, Consuelo Cháfer-Pericás

**Affiliations:** 1 Alzheimer’s Disease Research Group, Instituto de Investigación Sanitaria La Fe, 46026 Valencia, Spain; mariadelcarmen_pena@iislafe.es (C.P.-B.); lourdes_alvarez@iislafe.es (L.Á.-S.); gemma.garcia2@alumnos.uchceu.es (G.G.-L.); luis_raga@iislafe.es (L.R.); mar_pereto@iislafe.es (M.P.);; 2 Faculty of Mathematical Sciences, University of Valencia, 46100 Burjassot, Spain; angel.balaguer@uv.es; 3 Division of Neurology, Hospital Universitari I Politècnic La Fe, 46026 Valencia, Spain

**Keywords:** Alzheimer, lipidomics, plasma, subtypes, cognitive status

## Abstract

Alzheimer disease (AD) is a heterogeneous and complex disease in which different pathophysiological mechanisms are involved. This heterogenicity can be reflected in different atrophy patterns or clinical manifestations. Regarding biochemical pathways involved in early AD, lipid metabolism plays an important role; therefore, lipid levels have been evaluated as potential AD diagnosis biomarkers, and their levels could be related to different AD clinical manifestations. Therefore, the aim of this work is to study AD lipid profiles from early AD patients and evaluate their clinical significance. For this purpose, untargeted plasma lipidomic analysis was carried out in early AD patients (*n* = 31) diagnosed with cerebrospinal fluid (CSF) biomarkers. Cluster analysis was carried out to define early AD subgroups according to the lipid levels. Then, the clinical significance of each lipid profile subgroup was studied, analyzing differences for other variables (cognitive status, CSF biomarkers, medication, comorbidities, age, and gender). The cluster analysis revealed two different groups of AD patients. Cluster 1 showed higher levels of plasma lipids and better cognitive status than Cluster 2. However, no differences were found for the other variables (age, gender, medication, comorbidities, cholesterol, and triglycerides levels) between both groups. Plasma lipid levels could differentiate two early AD subgroups, which showed different cognitive statuses. However, further research with a large cohort and longitudinal study evaluating the clinical evolution of these patients is required. In general, it would involve a relevant advance in the knowledge of AD pathological mechanisms, potential treatments, and precision medicine.

## 1. Introduction

Alzheimer Disease (AD) is characterized clinically by a progressive deterioration in cognition and functionality over several years from preclinical stages, without cognitive impairment, until dementia stages, with severe cognitive and functional alterations [1]. However, the course of the pathology is complex and could be different among patients [2]. In general, memory loss is the most characteristic symptom, although other cognitive and functional domains can be affected (e.g., language, visuospatial ability, behavior, functional, and alterations) [3]. Few studies have focused on these different AD clinical aspects describing AD clinical subtypes [4]. First, previous studies identified different subtypes from neuropathology and neuroimaging results, specifically, brain atrophy and tau-related pathology distribution (typical, limbic-predominant, hippocampal-sparing AD, and minimal atrophy AD) [5]. In addition, a cerebrospinal fluid (CSF) proteomics study defined three AD subtypes according to their biochemical profile (hyperplasticity and increased β-Secretase 1 (BACE1) levels, innate immune activation, and blood–brain barrier dysfunction with low BACE1 levels) [6]. Also, an RNA sequencing study proposed three subtypes based on combinations of multiple dysregulated pathways such as tau-mediated neurodegeneration, amyloid-β neuroinflammation, synaptic signaling, immune activity, etc. [7]. Finally, recent research has focused on the clinical significance of these AD subtypes in terms of disease duration and progression rate [8].

However, several mechanisms such as tau hyperphosphorylation, amyloid accumulation, neuroinflammation, lipid dysregulation, oxidative stress, inflammation, and energy metabolism autophagy have an impact on clinical manifestations and disease progression [2,9]. In fact, the development of current clinical trials focused on different targets (tau, amyloid, neurotransmission, oxidative stress, inflammation, apolipoprotein E (ApoE), etc.) [10]. Furthermore, recent studies showed that lipid metabolism could play an important role in the development of the disease [11]. Actually, the apolipoprotein E genotype, related to lipid transport and metabolism, is one of the main AD risk factors [12,13,14].

Regarding lipid metabolism, the brain has a high lipid content, so lipid homeostasis impairment could be a relevant pathway in neurodegeneration [15]. In this sense, Chew et al. described the relationship between dyshomeostasis of brain lipids and AD through different pathophysiological mechanisms [16]. In addition, lipid rafts are required for the processing of amyloid precursor protein (APP), generating different amyloid peptides (e.g., Aβ42) and promoting the oligomerization of monomers to fibrils (e.g., amyloid plaques) [17]. In general, interactions between lipid metabolism and AD pathogenic mechanisms have been described; specifically, ceramides, cholesterol, and gangliosides showed a relationship with amyloid pathology, and also cholesterol showed a relationship with tau [18]. Also, a colocalization of lipids (e.g., cholesterol, ApoE) and amyloid plaques was observed in some studies [19,20]. Therefore, there is an increasing interest in the determination of lipids in different biological fluids (e.g., CSF, plasma) as potential AD biomarkers [21,22]. Some lipid panels have been postulated as potential biomarkers for the diagnosis and prediction of cognitive decline [23,24,25]. Among them, cholesterol or triglycerides are the most studied lipids in serum [26,27]. In general, higher levels have been related to cognitive decline risk [27], and they have been evaluated in case–control studies [28] or in normal elderly populations [27,29]. Specifically, McFarlane et al. found higher levels of total cholesterol and low-density lipoprotein (LDL) in mild cognitive impairment (MCI) than in cognitive normal and dementia groups, suggesting an increase and then the restorage of basal levels [28], while high-density lipoprotein (HDL) could be considered as a protective factor against cognitive impairment [27]. However, few lipidomics studies have focused on differential lipid metabolism among AD patients and their clinical status.

Therefore, the aim of this study is to describe early AD plasma lipid profiles and their relationship with the patient’s clinical status.

## 2. Results

### 2.1. Participants Description

Participants in this study were early AD patients and their clinical and demographic characteristics are summarized in (Table 1). Their median age was 70.5 years and 48% of participants were females. In general, the participants included in the study are late onset AD patients (LOAD) with the disease start disease from around 65 years old. Dyslipidemia and hypertension were the most common comorbidities, and statins and antihypertensives were the predominant used drugs. The median values for CSF Aβ42, t-Tau, and p-Tau were 508, 526, and 76 pg mL^−1^, respectively. Regarding cognitive status, the median score for CDR was 0.5, for RBANS.DM it was 60, for MMSE it was between 24 and 28, and for FAQ it was 5.

### 2.2. Clustering Analysis and Lipidomic Profile

**Figure 1:**
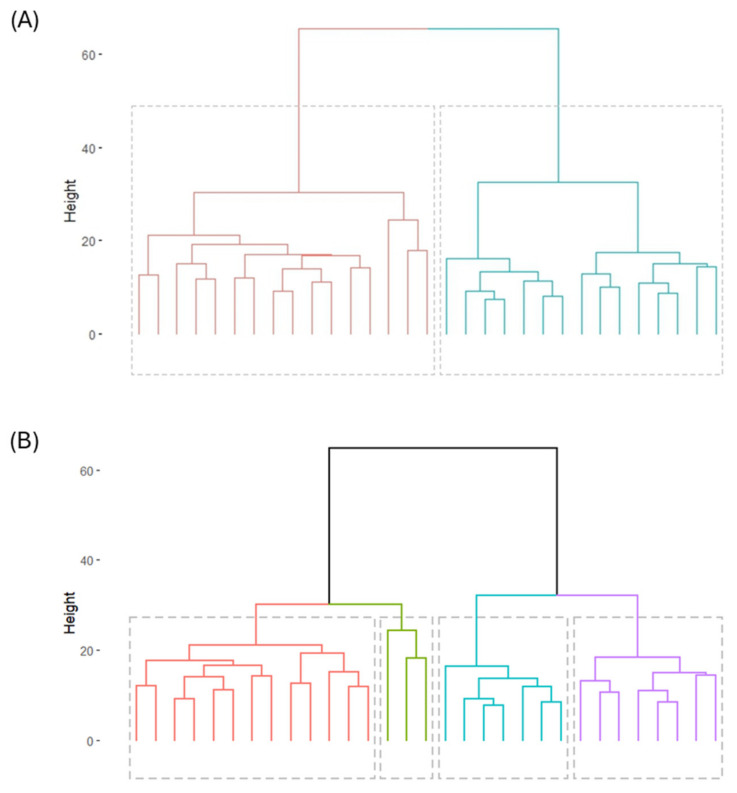
Dendrogram for hierarchical clustering for (**A**) two clusters and (**B**) four clusters. Each color represents one cluster.

**Figure 2:**
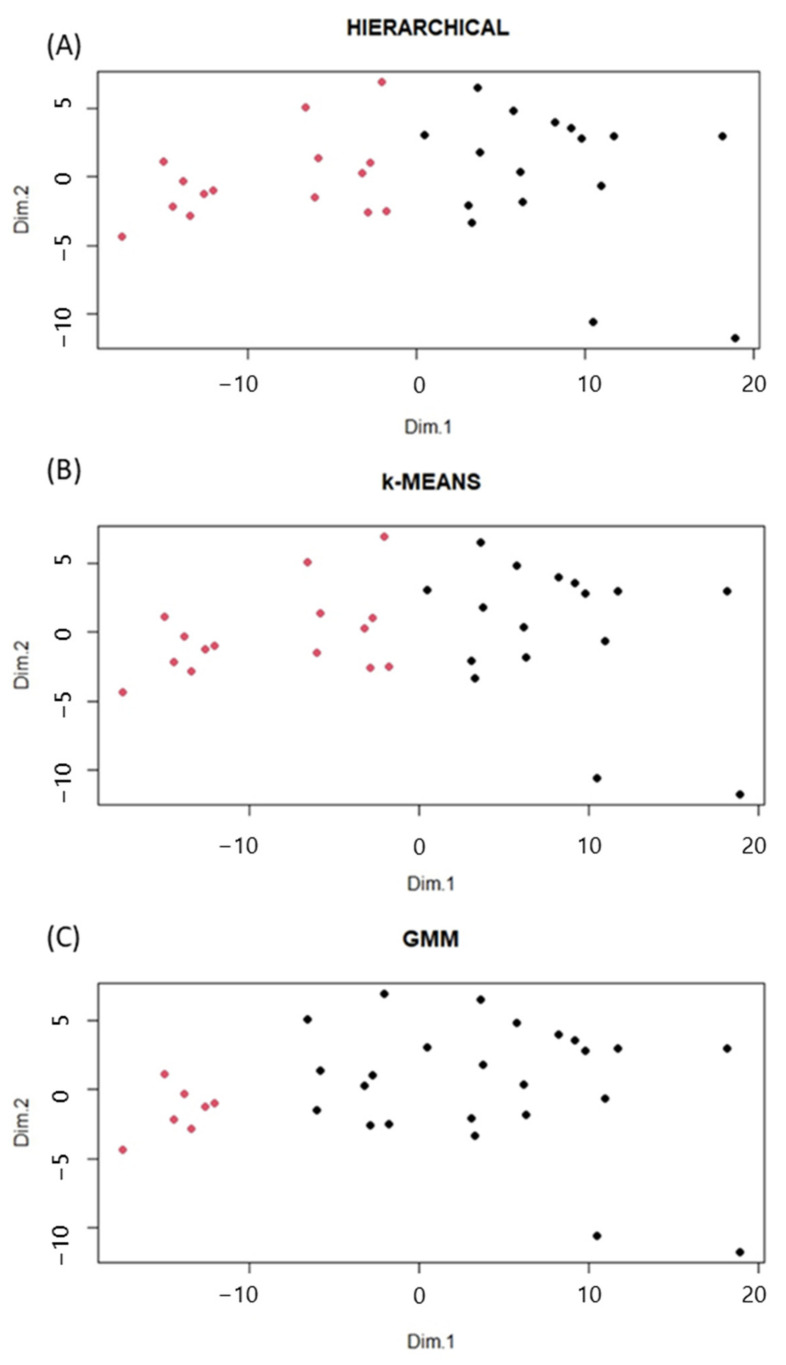
PCA scores plot representing the distribution of participants according to their plasma lipid profile in two components for each clustering model: (**A**) Hierarchical, (**B**) k-Means, and (**C**) Gaussian Mixture Model (black = Cluster 1, red = Cluster 2).

Three different non-supervised clustering methods were applied (Hierarchical, k-means, GMM), and the optimal number of clusters was four. However, as can be seen in Figure 1, the selected four groups are a subdivision from two bigger groups. From this observation, and together with the small sample size, it was decided to carry out the study for two clusters. Figure 2 represents the principal components distribution for each clustering model. The selected membership group is colored black for Cluster 1 and red for Cluster 2. 

Hierarchical and k-means models provided the same participants classification (cluster 1 (*n* = 16 participants), cluster 2 (*n* = 15)), while the GMM model provided a different participants classification (cluster 1 (*n* = 7) and cluster 2 (*n* = 24)). Therefore, the analysis obtained with hierarchical and k-means was selected, since the classification using the GMM model included a small group of 7 participants.

From the Hierarchical and k-means models, the obtained participants’ classification and their lipid profiles were analyzed. The plasma lipid levels grouped into families (cholesterol esters (CE); ceramides (Cer); diglycerols (DG); fatty acids (FA); lysophosphatidylcholines (LPC); lysophosphatidylethanolamines (LPE); monoglycerides (MG); phosphatidylcholines (PC); phosphatidylethanolamines (PE); phosphatidylinositols (PI); sphingomyelins (SM); and triglycerides (TG)) for each cluster are summarized in (Table 2). As can be seen, cluster 1 (*n* = 16) showed higher levels of lipids for most of the lipid families. 

**Figure 3:**
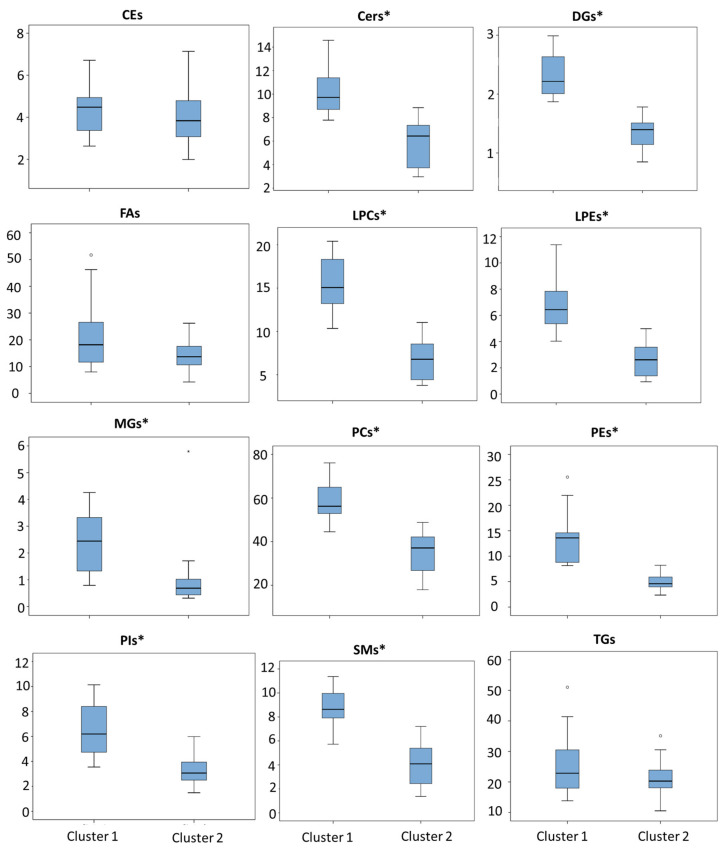
Box-plots representing levels of lipid families (CEs, Cers, DGs, FAs, LPCs, LPEs, MGs, PCs, PEs, PIs, SMs, and TGs) in both clusters. *: Statistically significant differences; o: atypical value.

Statistically significant differences were found for nine lipid families (sum of signals from individual lipids) between clusters: Cers (*p* < 0.001), DGs (*p* < 0.001), LPCs (*p* < 0.001), LPEs (*p* < 0.001), MGs (*p* < 0.001), PCs (*p* < 0.001), PEs (*p* < 0.001), PIs (*p* < 0.001), and SMs (*p* < 0.001); while CEs (*p* = 0.318), FAs (*p* = 0.188), and TGs (*p* = 0.338) did not show statistically significant differences between clusters (Figure 3).

Regarding univariate analysis, statistically significant differences were found for all the individual variables identified as DGs (2/2), LPEs (3/3), MGs (2/2), PEs (9/9), PIs (5/5), and SMs (12/12), and almost all the variables identified as Cers (14/16), LPCs (15/16), and PCs (63/73). However, only 2/4 CEs, 8/20 FAs, and 18/35 TGs showed statistically significant differences between clusters.

### 2.3. Clinical Significance of Lipid Profile

Participants from both clusters did not show statistically significant differences in age (*p* = 0.715), sex (*p* = 0.210), or educational level (*p* = 0.184); nor differences were found for comorbidities (dyslipidemia: *p* = 1.000; diabetes: *p* = 0.141; hypertension: *p* = 0.691), and drugs (statins: *p* = 0.691; fibrates: *p* = 0.238; morphics: *p* = 0.147; neuroleptics: *p* = 0.619; antidepressants: *p* = 1.000; antihypertensives: *p* = 0.561). In this sense, no differences were found for lipid-lowering drugs (*p* = 0.934). Moreover, both clusters were similar in ApoE genotype, considering allele ε4 carriers (*p* = 0.622, data available for n = 17).

For CSF biomarkers, no statistically significant differences were obtained between clusters (Aβ42, *p* = 0.928.; t-Tau, *p* = 0.185; p-Tau181, *p* = 0.098; Aβ40, *p* = 1.000; Aβ42/Aβ40, *p* = 0.352; Neurofilament light chain (NfL), *p* = 0.286; t-Tau/Aβ42, *p* = 0.108). In addition, no correlation was found for any lipid class with CSF AD biomarkers or age. 

Moreover, brain structural status evaluated using visual rating Medial temporal lobe atrophy (MTA) and Fazekas did not show differences between both clusters (MTA global *p* = 0.304; MTA right *p* = 0.617; MTA left *p* = 0.484; MTA sum *p* = 0.543; Fazekas *p* = 0.248).

**Figure 4:**
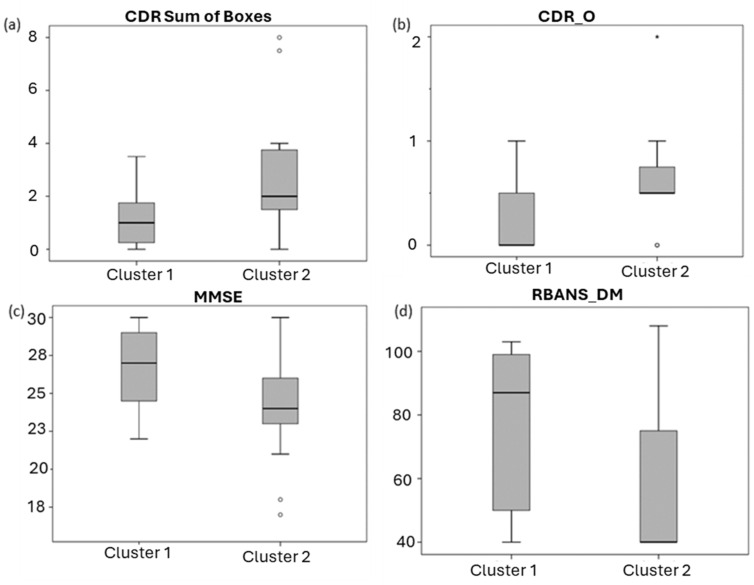
Boxplots representing neuropsychological assessment (**a**) CDR sum of boxes, (**b**) CDR Orientation (CDR.O), (**c**) MMSE, and (**d**) RBANS.DM) scores in each cluster. *: atypical value.

For cognitive status, some statistically significant differences were obtained between both clusters (MMSE, CDR sum of boxes, CDR.O, and RBANS.DM scores). Specifically, participants in cluster 1 showed higher levels of RBANS.DM (*p* = 0.008) and MMSE (*p* = 0.028) and lower scores for CDR sum boxes (*p* = 0.017) and CDR.O (*p* = 0.028) (see (Figure 4)).

**Figure 5:**
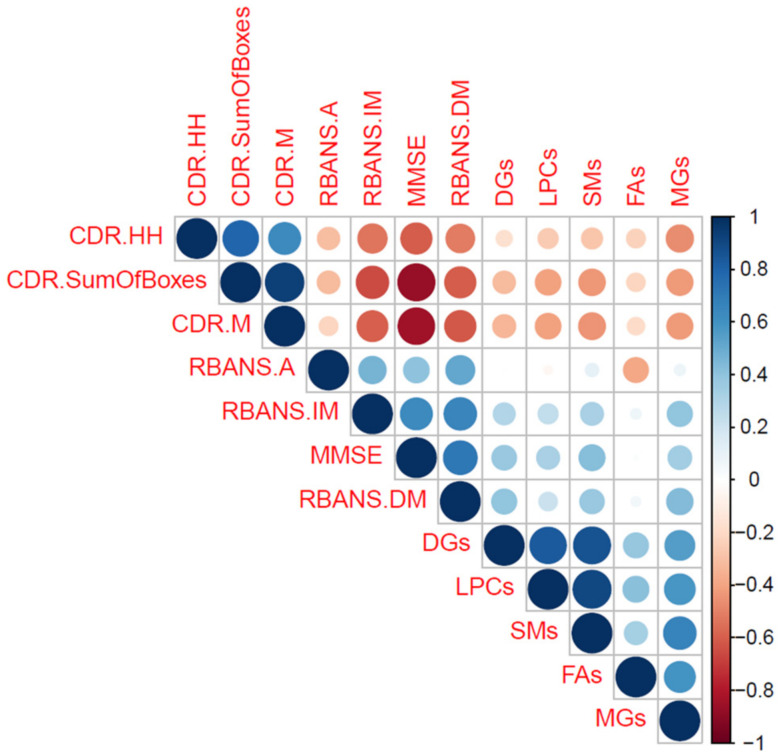
Correlation Plot. The size of the circles represents the strength of the correlation. The red color represents negative correlation, while blue represents positive correlations.

In addition, these neuropsychological scores showed a significant correlation with some lipid levels. Specifically, the CDR sum of boxes correlated negatively with LPCs, MGs, and SMs, while MMSE correlated positively with DGs, and SMs, and RBANS.DM with DGs, MGs, and SMs (see (Table 3 and Figure 5)). 

### 2.4. Lipid Profile for Progression

**Figure 6:**
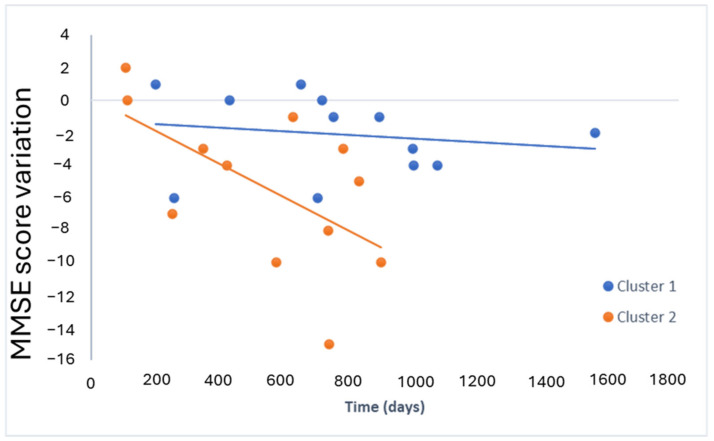
Cognitive decline measured by MMSE over time in both clusters.

The cognitive decline over time for both clusters is represented in (Figure 6). Specifically, it was evaluated the decline in MMSE score over time. 

The estimated values for the intercepts and slopes of each linear model, as well as the standard errors and *p*-values (Wald test), are shown in (Table 4).

As can be seen, only the model 2 slope was significant (*p*-value = 0.04), indicating the relationship between the MMSE score variation and time (days) in cluster 2.

Both models’ slopes were compared using the linear regression model including the interaction between the independent variable and the “factor” (cluster 1 or 2). The estimated values for the coefficients and the standard errors and *p*-values (Wald test) of this joint model are shown in (Table 5).

The Wald test results for the interaction coefficient indicated a statistically significant difference in slopes (*p*-value < 0.05), providing strong evidence for distinct relationships between the MMSE score variation and the time (days) for cluster 1 and cluster 2. Specifically, cluster 2 showed a faster progression than cluster 1.

## 3. Discussion

The AD complexity could be explained by the different pathophysiological pathways involved in its course. It is reflected in the heterogenicity of symptoms and differential progression. The identification of AD subtypes could be relevant in further treatment development. In this sense, it is interesting to evaluate the levels of peripheral biomarkers to identify different profiles in minimally invasive samples from AD patients. Among involved pathways, lipid metabolism impairment could play an important role in AD development. However, to our knowledge, this is the first study evaluating early AD patients’ subgroups according to their plasma lipidomic profile. Specifically, two patients’ subgroups were differentiated according to the levels of 197 lipids obtained with untargeted lipidomic analysis, followed by unsupervised clustering analysis. From these subgroups, the relationship with demographic and clinical variables was evaluated.

Regarding the plasma lipid profile in early AD patients, one group showed higher lipid levels for most of the lipid families compared to the other group. In general, the studies from the literature focused on the utility of lipids as biomarkers differentiating AD from non-AD patients, but not in AD subgroups and its clinical significance. In this sense, Agarwal et al. described the association between plasma lipids, polymorphisms in genes associated with cholesterol transport, and AD [26]. In addition, a previous study found differences in lipid families (DGs, LPEs, LPCs, MGs, and SMs,) between early AD and cognitively normal participants [30]. Moreover, Wood et al. described the heterogenicity of early onset AD and MCI patients with targeted lipidomics, differentiating three participants’ groups according to their levels of ethanolamine plasmalogens (PlsEs) and DGs. [31]. Specifically, they defined a subgroup with lower levels of PIsEs, a subgroup with higher levels of DGs, and a third group without altered levels of these lipids. However, the studies focused on AD lipid profiles are mainly related to cholesterol and triglyceride levels [32]. Other studies based on proteomic analyses found subtypes of AD with different metabolic pathways altered (e.g., hyperplasticity, innate immune activation, and blood–brain barrier dysfunction), revealing the heterogenicity among AD patients [6]. 

Regarding the clinical and demographic characteristics of the AD subgroups according to lipid levels, patients from both groups were similar in terms of age, sex, drugs, and comorbidities. However, previous studies found an association between lipid levels (cholesterol, triglycerides, sphingomyelins, and docosahexaenoic acid) and age or sex [33,34,35]. Specifically, Wong et al. reported higher levels of LDL, HDL, total cholesterol, SMs, and docosahexaenoic acid in females [35]. By contrast, Ma et al. did not find an association with sex for HDL, LDL, and total cholesterol, but described an association of these plasma lipids with age [34]. Moreover, Ancelin et al. described a relationship between lower levels of TGs and lower risk for AD in woman but not in men [33]. In addition, Lim et al. found a relationship between age and 197 plasma lipids, as well as between sex and 385 lipids [36]. They described 385 lipid species associated with gender including glycerophospholipids esterified with docosahexaenoic acid, which are associated with female sex.

For standard CSF AD biomarkers (Aβ42, t-Tau, p-Tau, and NfL), the present study did not find differences between both lipid-level subgroups. Similarly, Hu et al. did not find a relationship between plasma lipids (cholesterol, triglycerides) and plasma Aβ42 [37]. Nevertheless, a previous study carried out in ADNI and UPenn cohorts found negative associations between lipid compounds (ethanolamine plasmalogens and phosphatidylethanolamines) and CSF t-Tau and t-Tau/Aβ42 levels relating plasmalogens with AD [38], but that lipidomic study used a targeted methodology instead of an untargeted. Also, Sakr et al. found associations between plasma lipids (ether-glycerophospholipids, lyso-glycerophospholipids, free-fatty acids, cholesterol esters, and complex sphingolipids) and CSF pTau/Aβ42 ratio [39], but that study was adjusted by ApoE genotype, which could explain the discrepancy with our study. In fact, ApoE4 is closely related to lipid metabolism, and previous studies found a modulation of plasma lipid levels by the ApoE genotype [40]. However, in the present study, no significant differences were observed between both lipid-level groups for the ApoE genotype (ε4-carrier, ε4 non-carrier), which may be due to the small number of cases with these available data. Similarly, a study carried out in Apo ε2, ε3, and ε4 knock-in mice described slight differences between ε2 and ε4 compared to ε3 for some individual lipids (PI, PE, PC, Cer, and SM) without overall differences for glycerophospholipids [41]. In addition, a previous work found that different AD genetic variants (APP/presenilin 1 (PSEN1)/PSEN2 and triggering receptor expressed on myeloid cells 2 (TREM2)) showed differential metabolomic and lipidomic profiles [42]. However, Lim et al. found a weak association between APOE ε4 and plasma lipids [36]. Therefore, the influence of ApoE genotype on the plasma lipid profile should be better understood for further clinical applications. 

Evaluating the cognitive status, both lipid-level groups in the present work showed significant differences for some neurocognitive tests (MMSE, CDR sum of boxes, CDR.O, and RBANS.DM). These findings could suggest a relationship between cognitive status and plasma lipid levels. In general, higher levels for the lipid families studied (Cers, LPCs, LPEs, MGs, etc.) showed an association with better cognitive status. It could be explained by the fact that free lipids are necessary and functional in the organism. Probably, under pathological conditions, lipids could be recruited in lipid rafts, reducing their levels in biological fluids, increasing Aβ42 formation and aggregation, and worsening AD clinical manifestations. A previous review described the relationship between lipidomics and cognitive dysfunction [43]. Specifically, McFarlane et al. found higher levels of total cholesterol and LDL in MCI compared to controls [28]. In addition, Lee et al. proposed HDL as a protective factor against cognitive impairment; also, triglycerides were proposed by Yu et al. as a protective factor but only for men, while LDL levels were a protective factor for women [27,29]. Wood et al. found higher levels of DAG 34:2 and DAG 36:2 and lower levels of PlsE 40:6 in MCI and early onset AD patients with low scores for MMSE [31]. So, differential lipid profiles were found in patients related to their cognitive status. In general, the cited studies from the literature are not completely comparable to our study because they include other participants’ groups different from AD. In addition, the analyzed lipids were different, and they were determined by targeted methods. In the present study, significant correlations were found between SMs and some scores (MMSE, CDR sum of boxes, and RBANS.DM). The study from Mielke et al. found an association between lower lipid (Cer, SMs) serum levels and cross-sectional memory impairment [44]. These results point in the same direction as ours, where higher levels of SM are related to a better cognitive status. They also found that women with the highest levels of sphingomyelins had a reduced risk of AD, and that effect was most pronounced among APOE ε4 carriers [45]. In addition, we found other significant correlations between lipid families (DGs, MGs, and LPCs) and cognitive status. In this sense, Wood et al. observed increased levels of DAGs and MAGs in early AD [46]. Although we found a relationship between plasma lipid profile and cognitive impairment in AD patients, this study design did not allow us to define the causality. In other words, a relationship has been observed, but it is not possible to affirm that a deregulation in lipid metabolism leads to greater deterioration.

Regarding progression, the present results showed that AD patients with higher plasma lipid levels had slower progression in cognition impairment. In this sense, previous longitudinal studies showed a relationship between lipid levels and cognitive impairment progression [43]. Specifically, they described associations between higher plasma levels (SM, dihydrosphingomyelin (DHSM), SM/ceramide, and DHSM/dihydroceramides (DHCer) ratios) and less progression on the MMSE and ADAS-Cog tests [47]. In addition, Dakterzada et al. found that plasma neutral and ether-linked lipids were involved in the progression from MCI to AD dementia, suggesting the involvement of lipid-mediated antioxidant mechanisms in AD [48]. 

The main limitation of the present study was the small sample size. However, the participants were accurately classified as MCI due to AD using CSF biomarkers, constituting a homogeneous group. In addition, the ApoE genotype data were not available for all the cases, since it is a retrospective study, limiting the evaluation of this variable. Another limitation is that the follow-up data have been obtained retrospectively from clinical history, so the time for the evaluations is not homogeneous. Therefore, further research with a large sample and longitudinal study is required to validate the utility of these findings for AD subgroups and prognosis prediction.

## 4. Materials and Methods

### 4.1. Participants and Sample Collection

**Figure 7:**
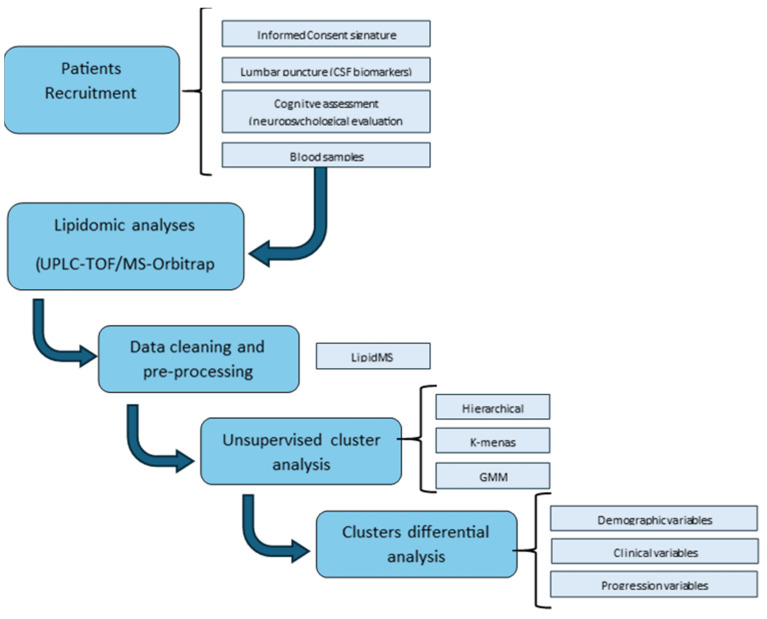
Scheme depicting the methodology.

The workflow followed in the study is described in Figure 7. Plasma samples from early AD patients between 50 and 80 years old were collected from the Neurology Service at the University and Polytechnic Hospital La Fe (Valencia, Spain). The available sample size was *n* = 30 since it is a retrospective study. Nevertheless, previous studies carried out with similar sample size showed satisfactory results [49,50]. Participants were diagnosed following the National Institute on Aging and the Alzheimer’s Association (NIA-AA) criteria [51]. Briefly, they have impaired CSF biomarkers (Aβ42, total Tau (t-Tau), phosphorylated Tau (p-Tau)) and mild cognitive impairment without altered daily living activities established using a neuropsychological evaluation. Clinical Dementia Rating (CDR) includes the following domains: memory (M), orientation (O), Judgment and Problem Solving (JPS), Community Affairs (CA), Home and Hobbies (HH), and Personal Care (PC). The Repeatable Battery for the Assessment of Neuropsychological Status (RBANS) includes the following domains: Delayed Memory (DM), Immediate Memory (IM), Visuospatial/Constructional (V/C:), Language (L), Attention (A) [52], Mini-Mental State Examination (MMSE) [53], Functionality Assessment Questionnaire (FAQ) [54], and Alzheimer’s Disease Cooperative Study—Activities of Daily Living for Mild Cognitive Impairment (ADCS-MCI-ADL) [55]. In addition, a second neuropsychological evaluation was carried out for some patients at 400–1600 days from diagnosis.

The study protocol was approved by the Ethics Committee (CEIC) of the Health Research Institute La Fe (Valencia, Spain) (2019/0105). 

### 4.2. Lipidomic Analysis

Untargeted lipidomic analysis was carried out as described by Peña-Bautista et al. [30]. Briefly, plasma samples were treated with cold isopropanol and centrifuged, and an internal standard (IS) (17:0 LPC, d18:1/17:0 SM, and 17:0 PE) was added. Then, the supernatant was analyzed using ultra-performance liquid chromatography coupled to time-of-flight mass spectrometry (UPLC-TOF/MS-Orbitrap QExactive Plus MS) following the normalized protocol from the Analytical Unit in Health Research Unit La Fe (Valencia, Spain). Briefly, it was carried out in an Acquity UPLC CSH C18 column (100 × 2.1 mm, 1.7 μm) from Waters and the mobile phase was acetonitrile/water (60:40) and isopropyl alcohol/acetonitrile (90:10) with formic acid 10 mM for the positive ionization mode and acetic acid 10 mM for the negative.

Data obtained from the untargeted lipidomic analyses were processed with the LipidMS R package (version 4.3.2) [56]. After that, the dataset was filtered, corrected, normalized, and annotated before statistical analyses [30].

### 4.3. Statistical Analysis

Unsupervised cluster analysis was carried out with the lipidic variables obtained from lipidomic analysis using the package “mclust” from software R (v 4.3.1) after data standardization using of the scale function. Specifically, three methods were assessed (Hierarchical, k-means, and Gaussian Mixture model (GMM)). Then, differences between obtained clusters were analyzed using a Mann–Whitney and Chi-Square for quantitative and qualitative variables, respectively, using SPSS v23 (SPSS, Inc., Chicago, IL, USA). Also, lipid level differences between clusters were analyzed using lipid families (sum of signals obtained for individual lipids in each family). Correlations were analyzed using a Pearson correlation. Statistical significance was defined as *p* value < 0.05.

Two linear models (one for each cluster) were developed for the study of progression including “MMSE score variation” as the dependent variable and “Time (days)” as the independent variable using R Studio software (version 4.3.2). In addition, a linear regression model was built including the interaction between the independent variable and a categorical variable “factor” that informs the clusters (1 or 2) of the data (y = intercept + slope x + interaction coefficient (x:factor)) to compare both models slopes. The inclusion of the interaction term (x:factor) allowed the evaluation of slope differences between both models, to compare the progression between both clusters. 

## 5. Conclusions

The lipid plasma profile could provide some relevant information for the characterization of AD patients. Mainly, two different early AD patients’ subgroups were distinguished according to plasma lipid profiles. In general, higher lipids levels showed significant association with better cognitive status and lower decline over time, defined by a complete neuropsychological evaluation. These preliminary results could help in future studies, in which the stratification of patients would be required to access specific clinical trials. However, further research with a high number of patients and longitudinal studies are required to validate these results.

## Figures and Tables

**Table 1:** Demographic and clinical characteristics of participants.

		Participants (*n* = 31)
Age (years) (median (IQR))		71 (68,74)
Sex (Female, n (%))		16 (48%)
Educational level (n, %)	Primary	11 (36.7%)
	Secondary	12 (38.7%)
	Universitary	7 (22.6%)
Drugs (n, %)	Statins	15 (48,4)
	Fibrates	4 (12.9)
	Benzodiazepines	5 (16.1%)
	Antidepressants	2 (6.5%)
	Antihypertensives	11 (35.5%)
Comorbidities (n, %)	Dislipidemia	14 (45.2%)
	Diabetes	2 (6.5%)
	Hypertension	11 (35.5%)
	Heart Disease	0 (0%)
	Cerebrovascular disease	0 (0%)
Smoke status (Yes) (n, %)	4 (12.9%)	
Alcohol (n, %)	2 (6.5%)	
Depression (n, %)	7 (22.6%)	
Anxiety (n, %)	4 (12.9%)	
ApoE4 carrier (n, %)	13 (76%) *	
CSF Amyloid-β42 (Aβ42) (pg mL^−1^) (median (IQR))	508 (436,675)	
CSF t-Tau (pg mL^−1^) (median (IQR))	526 (341,733)	
CSF p-Tau (pg mL^−1^) (median (IQR))	76 (57,105)	
CSF Amyloid-β40 (Aβ40) (pg mL^−1^)	10292 (5959,12464)	
Aβ42/Aβ40 (median (IQR))	0.06 (0.05,0.10)	
Neurofilament light chain (NfL) (pg mL^−1^) (median (IQR))	818 (550,1442)	
t-Tau/Aβ42 (median (IQR))	0.94 (0.68,1.30)	
CDR (score) (median (IQR))	0.5 (0.5,0.5)	
MMSE (score) (median (IQR))	26 (24,28)	
RBANS.DM (score) (median (IQR))	60 (40,91)	
FAQ (score) (median (IQR))	5 (1,8)	

CSF: cerebrospinal fluid; CDR: Clinical Dementia Rating; MMSE: Mini-Mental State Examination; FAQ: Functionality Assessment Questionnaire; RBANS: Repeatable Battery for Assessment of Neuropsychological Status; DM: Delayed memory. FAQ: Functional Activities Questionnaire; * Data available from 17 patients.

**Table 2:** Plasma levels obtained for the different lipid classes in each cluster.

Lipid Family	Cluster 1 (*n* = 16)		Cluster 2 (*n* = 15)		*p* Value (Mann–Whitney)
	Mean (SD) (a.u.)	Median (IQR) (a.u.)	Mean (SD) (a.u.)	Median (IQR) (a.u.)	
CEs	4.33 (1.08)	4.48 (3.37,4.95)	3.94 (1.38)	3.84 (3.05,5.17)	0.318
Cers	10.28 (2.05)	9.72 (8.65,11.649)	5.71 (2.15)	6.43 (3.59,7.77)	0.000 *
DGs	2.33 (0.38)	2.22 (2.00,2.69)	1.34 (0.28)	1.40 (1.06,1.55)	0.000 *
FAs	22.05 (13.68)	18.16 (11.00,27.23)	14.26 (5.84)	13.68 (10.65,19.50)	0.188
LPCs	15.53 (3.03)	15.06 (12.82,18.40)	6.80 (2.57)	6.78 (4.35,9.79)	0.000 *
LPEs	6.76 (1.86)	6.44 (5.31,7.97)	2.65 (1.33)	2.61 (1.36,4.00)	0.000 *
MGs	2.37 (1.13)	2.44 (1.19,3.44)	1.06 (1.36)	0.68 (0.40,1,12)	0.000 *
PCs	58.29 (8.17)	56.25 (52.65,65.79)	34.80 (9.23)	37.11 (25.56,42.34)	0.000 *
PEs	13.21 (4.99)	13.60 (8.64,14.59)	4.89 (1.59)	4.59 (3.82,6.40)	0.000 *
PIs	6.63 (2.03)	6.20 (4.70,8.55)	3.33 (1.34)	3.07 (2.48,3.98)	0.000 *
SMs	8.75 (1.43)	8.63 (7.89,10.02)	4.05 (1.85)	4.09 (2.37,6.01)	0.000 *
TGs	25.60 (10.16)	22.87 (17.90,31.90)	21.36 (6.24)	20.29 (17.84,26.15)	0.338

CE: Cholesterol esters; Cer: Ceramides; DG: Diglycerols; FA: Fatty acids; LPC: Lyso phosphatidylcholines; LPE: Lysophosphatidylethanolamines; MG: Monoglycerides; PC: Phosphatidylcholines; PE: Phosphatidylethanolamines; PI: Phosphatidylinositols; SM: Sphingomyelins; TG: Triglycerides. IQR: interquartile range; a.u. arbitrary units. * *p*-value < 0.05.

**Table 3:** Correlations evaluation between lipid classes (DGs, FAs, LPCs, MGs, and SMs) levels and neurocognitive status (CDR, MMSE, and RBANS).

	SUMBOX CDR	CDRM	CDRADA	MMSE	RBANS.MI	RBANS.A	RBANS.DM
DGs (PCC (*p* value))	−0.312 (0.14)	−0.330 (0.12)	−0.161 (0.45)	0.378 (0.039) *	0.298 (0.11)	−0.002 (0.99)	0.391 (0.033) *
FAs (PCC (*p* value))	−0.211 (0.32)	−0.190 (0.38)	−0.221 (0.30)	0.011 (0.96)	0.069 (0.72)	−0.387 (0.034) *	0.059 (0.76)
LPCs (PCC (*p* value))	−0.407 (0.049) *	−0.410 (0.047) *	−0.251 (0.24)	0.328 (0.08)	0.248 (0.19)	−0.043 (0.82)	0.213 (0.26)
MGs (PCC (*p* value))	−0.427 (0.037) *	−0.423 (0.039) *	−0.463 (0.023) *	0.350 (0.06)	0.393 (0.032) *	0.077 (0.69)	0.431 (0.018) *
SMs (PCC (*p* value))	−0.437 (0.033) *	−0.449 (0.028) *	−0.275 (0.19)	0.420 (0.021) *	0.324 (0.08)	0.108 (0.57)	0.371 (0.044) *

PCC: Pearson correlation coefficient. *: *p*-value < 0.05.

**Table 4:** Summary of regression models 1 and 2, corresponding to clusters 1 and 2, respectively.

	Estimate	Standard Error	*p*-Value
Intercept (model 1)	−1.2	1.8	0.4990
Slope (model 1)	−0.001	0.002	0.6040
Intercept (model 2)	0	3	0.9591
Slope (model 2)	−0.010	0.004	0.0436 *

*: *p* value < 0.05.

**Table 5:** Summary of the regression joint model.

	Estimate	Standard Error	*p*-Value
Intercept	−0.5	1.6	0.7494
Slope (x)	−0.002	0.002	0.3666
Interaction Coefficient (x:factor)	−0.007	0.002	0.0021 **

**: *p* value < 0.01.

## Data Availability

Data are contained within the article.

## Abbreviations

Aβ42	Amyloid β42
AD	Alzheimer disease
ADCS-MCI-ADL	Alzheimer’s Disease Cooperative Study-Activities of Daily Living for Mild Cognitive Impairment
ApoE	Apolipoprotein E
APP	Amyloid precursor peptide
BACE1	β-Secretase 1
CDR	Clinical Dementia Rating
CE	Cholesterol ester
Cer	Ceramide
CSF	Cerebrospinal fluid
CEIC	Ethics Committee
DG	diglycerols
DHCer	dihydroceramides
DHSM	dihydrosphingomyelin
FA	fatty acids
FAQ	Functionality Assessment Questionnaire
GMM	Gaussian Mixture model
HDL	high-density lipoprotein
LDL	density lipoprotein
LPC	Lysophosphatidylcholines
LPE	Lysophosphatidylethanolamines
MCI	Mild cognitive impairment
MG	Monoglycerides
MMSE	Mini-Mental State Examination
MTA	Medial temporal lobe atrophy
NIA-AA	National Institute on Aging and the Alzheimer’s Association
NfL	Neurofilament light chain
PC	phosphatidylcholines
PE	phosphatidylethanolamines
PI	phosphatidylinositols
PIsEs	Ethanolamine plasmalogens
PSEN	presenilin
p-Tau	phosphorylated Tau
RBANS	Repeatable Battery for the Assessment of Neuropsychological Status
SM	sphingomyelins
TG	triglycerides
TREM2	Triggering receptor expressed on myeloid cells 2
t-Tau	total Tau
UPLC-TOF/MS	ultra-performance liquid chromatography coupled to time-of-flight mass spectrometry

## References

[1] Aisen P.S., Cummings J., Jack C.R., Morris J.C., Sperling R., Frölich L., Jones R.W., Dowsett S.A., Matthews B.R., Raskin J. (2017). On the Path to 2025: Understanding the Alzheimer’s Disease Continuum. Alzheimers Res. Ther..

[2] Duara R., Barker W. (2022). Heterogeneity in Alzheimer’s Disease Diagnosis and Progression Rates: Implications for Therapeutic Trials. Neurotherapeutics.

[3] Ferrari C., Sorbi S. (2021). The Complexity of Alzheimer’s Disease: An Evolving Puzzle. Physiol. Rev..

[4] Petersen R.C. (1998). Clinical Subtypes of Alzheimer’s Disease. Dement. Geriatr. Cogn. Disord..

[5] Ferreira D., Nordberg A., Westman E. (2020). Biological Subtypes of Alzheimer Disease. Neurology.

[6] Tijms B.M., Gobom J., Reus L., Jansen I., Hong S., Dobricic V., Kilpert F., ten Kate M., Barkhof F., Tsolaki M. (2020). Pathophysiological Subtypes of Alzheimer’s Disease Based on Cerebrospinal Fluid Proteomics. Brain.

[7] Neff R.A., Wang M., Vatansever S., Guo L., Ming C., Wang Q., Wang E., Horgusluoglu-Moloch E., Song W., Li A. (2021). Molecular Subtyping of Alzheimer’s Disease Using RNA Sequencing Data Reveals Novel Mechanisms and Targets. Sci. Adv..

[8] Jellinger K.A. (2020). Pathobiological Subtypes of Alzheimer Disease. Dement. Geriatr. Cogn. Disord..

[9] Calabrò M., Rinaldi C., Santoro G., Crisafulli C. (2021). The Biological Pathways of Alzheimer Disease: A Review. AIMS Neurosci..

[10] Cummings J., Zhou Y., Lee G., Zhong K., Fonseca J., Cheng F. (2023). Alzheimer’s Disease Drug Development Pipeline: 2023. Alzheimer’s Dement. Transl. Res. Clin. Interv..

[11] García-Viñuales S., Sciacca M.F.M., Lanza V., Santoro A.M., Grasso G., Tundo G.R., Sbardella D., Coletta M., Grasso G., La Rosa C. (2021). The Interplay between Lipid and Aβ Amyloid Homeostasis in Alzheimer’s Disease: Risk Factors and Therapeutic Opportunities. Chem. Phys. Lipids.

[12] Koutsodendris N., Nelson M.R., Rao A., Huang Y. (2022). Apolipoprotein E and Alzheimer’s Disease: Findings, Hypotheses, and Potential Mechanisms. Annu. Rev. Pathol. Mech. Dis..

[13] Huang Y., Mahley R.W. (2014). Apolipoprotein E: Structure and Function in Lipid Metabolism, Neurobiology, and Alzheimer’s Diseases. Neurobiol. Dis..

[14] Rudge J.D. (2022). A New Hypothesis for Alzheimer’s Disease: The Lipid Invasion Model. J. Alzheimers Dis. Rep..

[15] Kao Y.-C., Ho P.-C., Tu Y.-K., Jou I.-M., Tsai K.-J. (2020). Lipids and Alzheimer’s Disease. Int. J. Mol. Sci..

[16] Chew H., Solomon V.A., Fonteh A.N. (2020). Involvement of Lipids in Alzheimer’s Disease Pathology and Potential Therapies. Front. Physiol..

[17] Lai A.Y., McLaurin J. (2011). Mechanisms of Amyloid-Beta Peptide Uptake by Neurons: The Role of Lipid Rafts and Lipid Raft-Associated Proteins. Int. J. Alzheimers Dis..

[18] Yin F. (2023). Lipid Metabolism and Alzheimer’s Disease: Clinical Evidence, Mechanistic Link and Therapeutic Promise. FEBS J..

[19] Benseny-Cases N., Klementieva O., Cotte M., Ferrer I., Cladera J. (2014). Microspectroscopy (ΜFTIR) Reveals Co-Localization of Lipid Oxidation and Amyloid Plaques in Human Alzheimer Disease Brains. Anal. Chem..

[20] Burns M.P., Noble W.J., Olm V., Gaynor K., Casey E., LaFrancois J., Wang L., Duff K. (2003). Co-Localization of Cholesterol, Apolipoprotein E and Fibrillar Aβ in Amyloid Plaques. Mol. Brain Res..

[21] Byeon S.K., Madugundu A.K., Jain A.P., Bhat F.A., Jung J.H., Renuse S., Darrow J., Bakker A., Albert M., Moghekar A. (2021). Cerebrospinal Fluid Lipidomics for Biomarkers of Alzheimer’s Disease. Mol. Omics.

[22] Casas-Fernández E., Peña-Bautista C., Baquero M., Cháfer-Pericás C. (2022). Lipids as Early and Minimally Invasive Biomarkers for Alzheimer’s Disease. Curr. Neuropharmacol..

[23] Fiandaca M.S., Zhong X., Cheema A.K., Orquiza M.H., Chidambaram S., Tan M.T., Gresenz C.R., FitzGerald K.T., Nalls M.A., Singleton A.B. (2015). Plasma 24-Metabolite Panel Predicts Preclinical Transition to Clinical Stages of Alzheimer’s Disease. Front. Neurol..

[24] Ma Y., Shen X., Xu W., Huang Y., Li H., Tan L., Tan C., Dong Q., Tan L., Yu J. (2020). A Panel of Blood Lipids Associated with Cognitive Performance, Brain Atrophy, and Alzheimer’s Diagnosis: A Longitudinal Study of Elders without Dementia. Alzheimer’s Dement. Diagn. Assess. Dis. Monit..

[25] Zhang X., Liu W., Zan J., Wu C., Tan W. (2020). Untargeted Lipidomics Reveals Progression of Early Alzheimer’s Disease in APP/PS1 Transgenic Mice. Sci. Rep..

[26] Agarwal M., Khan S. (2020). Plasma Lipids as Biomarkers for Alzheimer’s Disease: A Systematic Review. Cureus.

[27] Lee J., Lee S., Min J., Min K. (2021). Association between Serum Lipid Parameters and Cognitive Performance in Older Adults. J. Clin. Med..

[28] McFarlane O., Kozakiewicz M., Kędziora-Kornatowska K., Gębka D., Szybalska A., Szwed M., Klich-Rączka A. (2020). Blood Lipids and Cognitive Performance of Aging Polish Adults: A Case-Control Study Based on the PolSenior Project. Front. Aging Neurosci..

[29] Yu Y., Yan P., Cheng G., Liu D., Xu L., Yang M., Xu H., Cheng X., Lian P., Zeng Y. (2023). Correlation between Serum Lipid Profiles and Cognitive Impairment in Old Age: A Cross-Sectional Study. Gen. Psychiatr..

[30] Peña-Bautista C., Álvarez-Sánchez L., Roca M., García-Vallés L., Baquero M., Cháfer-Pericás C. (2022). Plasma Lipidomics Approach in Early and Specific Alzheimer’s Disease Diagnosis. J. Clin. Med..

[31] Wood P.L., Locke V.A., Herling P., Passaro A., Vigna G.B., Volpato S., Valacchi G., Cervellati C., Zuliani G. (2016). Targeted Lipidomics Distinguishes Patient Subgroups in Mild Cognitive Impairment (MCI) and Late Onset Alzheimer’s Disease (LOAD). BBA Clin..

[32] Sabbagh M., Zahiri H.R., Ceimo J., Cooper K., Gaul W., Connor D., Sparks D.L. (2005). Is There a Characteristic Lipid Profile in Alzheimer’s Disease?. J. Alzheimer’s Dis..

[33] Ancelin M.-L., Ripoche E., Dupuy A.-M., Barberger-Gateau P., Auriacombe S., Rouaud O., Berr C., Carrière I., Ritchie K. (2013). Sex Differences in the Associations Between Lipid Levels and Incident Dementia. J. Alzheimer’s Dis..

[34] Ma R.M., Li G.G., Ding Y.W., Lyu J., Shao C.Q., Liu J.Z., Liu J., Zhang G.J. (2022). Correlation of Serum Lipids Levels of Alzheimer’s Disease Patients with Sex, Age and Apolipoprotein E Gene Polymorphism. Zhonghua Yu Fang Yi Xue Za Zhi.

[35] Wong M.W.K., Braidy N., Pickford R., Vafaee F., Crawford J., Muenchhoff J., Schofield P., Attia J., Brodaty H., Sachdev P. (2019). Plasma Lipidome Variation during the Second Half of the Human Lifespan Is Associated with Age and Sex but Minimally with BMI. PLoS ONE.

[36] Lim W.L.F., Huynh K., Chatterjee P., Martins I., Jayawardana K.S., Giles C., Mellett N.A., Laws S.M., Bush A.I., Rowe C.C. (2020). Relationships Between Plasma Lipids Species, Gender, Risk Factors, and Alzheimer’s Disease. J. Alzheimer’s Dis..

[37] Hu N., Gao L., Jiang Y., Wei S., Shang S., Chen C., Dang L., Wang J., Huo K., Deng M. (2020). The Relationship between Blood Lipids and Plasma Amyloid Beta Is Depend on Blood Pressure: A Population-Based Cross-Sectional Study. Lipids Health Dis..

[38] Kling M.A., Goodenowe D.B., Senanayake V., MahmoudianDehkordi S., Arnold M., Massaro T.J., Baillie R., Han X., Leung Y., Saykin A.J. (2020). Circulating Ethanolamine Plasmalogen Indices in Alzheimer’s Disease: Relation to Diagnosis, Cognition, and CSF Tau. Alzheimer’s Dement..

[39] Sakr F., Dyrba M., Bräuer A., Teipel S. (2022). Association of Lipidomics Signatures in Blood with Clinical Progression in Preclinical and Prodromal Alzheimer’s Disease. J. Alzheimer’s Dis..

[40] Wong M.W.K., Braidy N., Crawford J., Pickford R., Song F., Mather K.A., Attia J., Brodaty H., Sachdev P., Poljak A. (2019). APOE Genotype Differentially Modulates Plasma Lipids in Healthy Older Individuals, with Relevance to Brain Health. J. Alzheimer’s Dis..

[41] Sharman M.J., Shui G., Fernandis A.Z., Lim W.L.F., Berger T., Hone E., Taddei K., Martins I.J., Ghiso J., Buxbaum J.D. (2010). Profiling Brain and Plasma Lipids in Human APOE Ε2, Ε3, and Ε4 Knock-in Mice Using Electrospray Ionization Mass Spectrometry. J. Alzheimer’s Dis..

[42] Novotny B.C., Fernandez M.V., Wang C., Budde J.P., Bergmann K., Eteleeb A.M., Bradley J., Webster C., Ebl C., Norton J. (2023). Metabolomic and Lipidomic Signatures in Autosomal Dominant and Late-onset Alzheimer’s Disease Brains. Alzheimer’s Dement..

[43] Chakraborty A., Praharaj S.K., Prabhu R.V.K., Prabhu M.M. (2020). Lipidomics and Cognitive Dysfunction—A Narrative Review. Turk. J. Biochem..

[44] Mielke M.M., Bandaru V.V.R., Haughey N.J., Rabins P.V., Lyketsos C.G., Carlson M.C. (2010). Serum Sphingomyelins and Ceramides Are Early Predictors of Memory Impairment. Neurobiol. Aging.

[45] Mielke M.M., Haughey N.J., Han D., An Y., Bandaru V.V.R., Lyketsos C.G., Ferrucci L., Resnick S.M. (2017). The Association Between Plasma Ceramides and Sphingomyelins and Risk of Alzheimer’s Disease Differs by Sex and APOE in the Baltimore Longitudinal Study of Aging. J. Alzheimer’s Dis..

[46] Wood P.L., Medicherla S., Sheikh N., Terry B., Phillipps A., Kaye J.A., Quinn J.F., Woltjer R.L. (2015). Targeted Lipidomics of Fontal Cortex and Plasma Diacylglycerols (DAG) in Mild Cognitive Impairment and Alzheimer’s Disease: Validation of DAG Accumulation Early in the Pathophysiology of Alzheimer’s Disease. J. Alzheimer’s Dis..

[47] Mielke M.M., Haughey N.J., Bandaru V.V.R., Weinberg D.D., Darby E., Zaidi N., Pavlik V., Doody R.S., Lyketsos C.G. (2011). Plasma Sphingomyelins Are Associated with Cognitive Progression in Alzheimer’s Disease. J. Alzheimer’s Dis..

[48] Dakterzada F., Jové M., Huerto R., Carnes A., Sol J., Pamplona R., Piñol-Ripoll G. (2023). Changes in Plasma Neutral and Ether-Linked Lipids Are Associated with The Pathology and Progression of Alzheimer’s Disease. Aging Dis..

[49] Tremblay-Franco M., Canlet C., Carriere A., Nakhle J., Galinier A., Portais J.-C., Yart A., Dray C., Lu W.-H., Bertrand Michel J. (2024). Integrative Multimodal Metabolomics to Early Predict Cognitive Decline Among Amyloid Positive Community-Dwelling Older Adults. J. Gerontol. A Biol. Sci. Med. Sci..

[50] Bredesen D.E. (2015). Metabolic Profiling Distinguishes Three Subtypes of Alzheimer’s Disease. Aging.

[51] Jack C.R., Bennett D.A., Blennow K., Carrillo M.C., Dunn B., Haeberlein S.B., Holtzman D.M., Jagust W., Jessen F., Karlawish J. (2018). NIA-AA Research Framework: Toward a Biological Definition of Alzheimer’s Disease. Alzheimer’s Dement..

[52] Randolph C., Tierney M.C., Mohr E., Chase T.N. (1998). The Repeatable Battery for the Assessment of Neuropsychological Status (RBANS): Preliminary Clinical Validity. J. Clin. Exp. Neuropsychol..

[53] Folstein M.F., Folstein S.E., McHugh P.R. (1975). “Mini-Mental State”: A practical method for grading the cognitive state of patients for the clinician. J. Psychiatr. Res..

[54] Pfeffer R.I., Kurosaki T.T., Harrah C.H., Chance J.M., Filos S. (1982). Measurement of Functional Activities in Older Adults in the Community. J. Gerontol..

[55] Galasko D., Bennett D., Sano M., Ernesto C., Thomas R., Grundman M., Ferris S. (1997). An Inventory to Assess Activities of Daily Living for Clinical Trials in Alzheimer’s Disease. Alzheimer Dis. Assoc. Disord..

[56] Alcoriza-Balaguer M.I., García-Cañaveras J.C., López A., Conde I., Juan O., Carretero J., Lahoz A. (2019). LipidMS: An R Package for Lipid Annotation in Untargeted Liquid Chromatography-Data Independent Acquisition-Mass Spectrometry Lipidomics. Anal. Chem..

